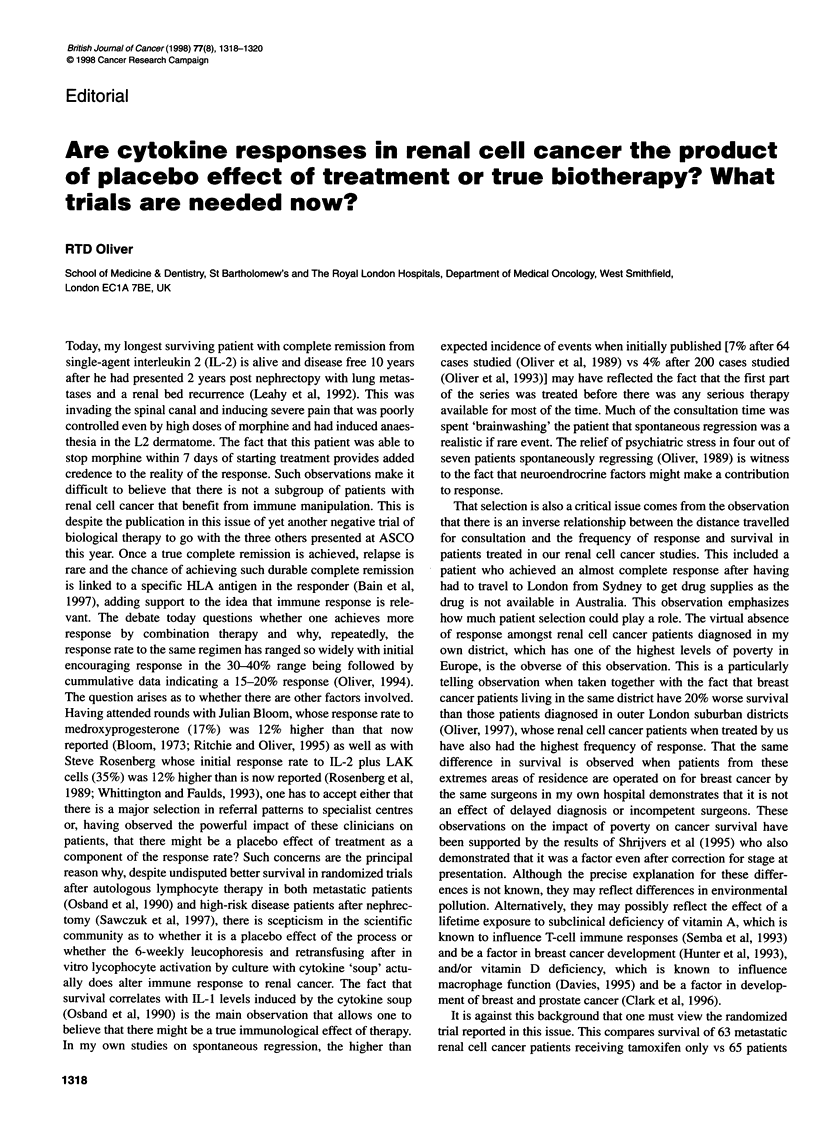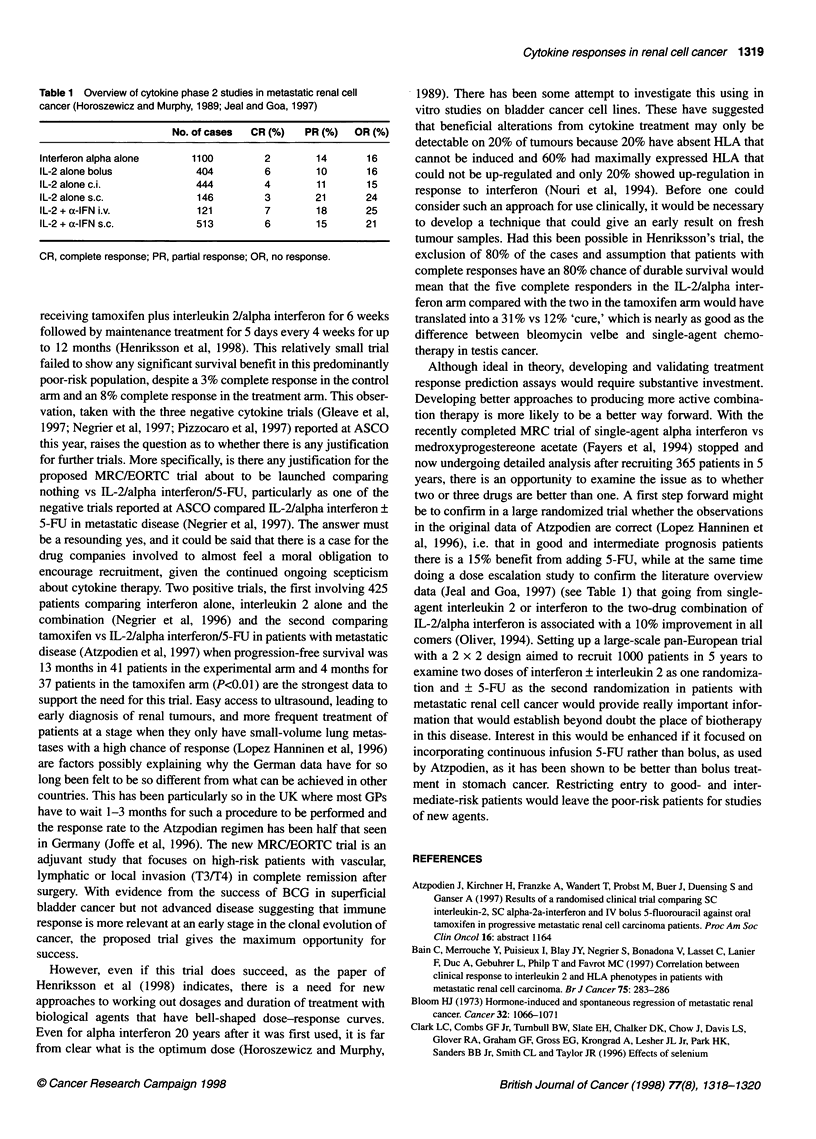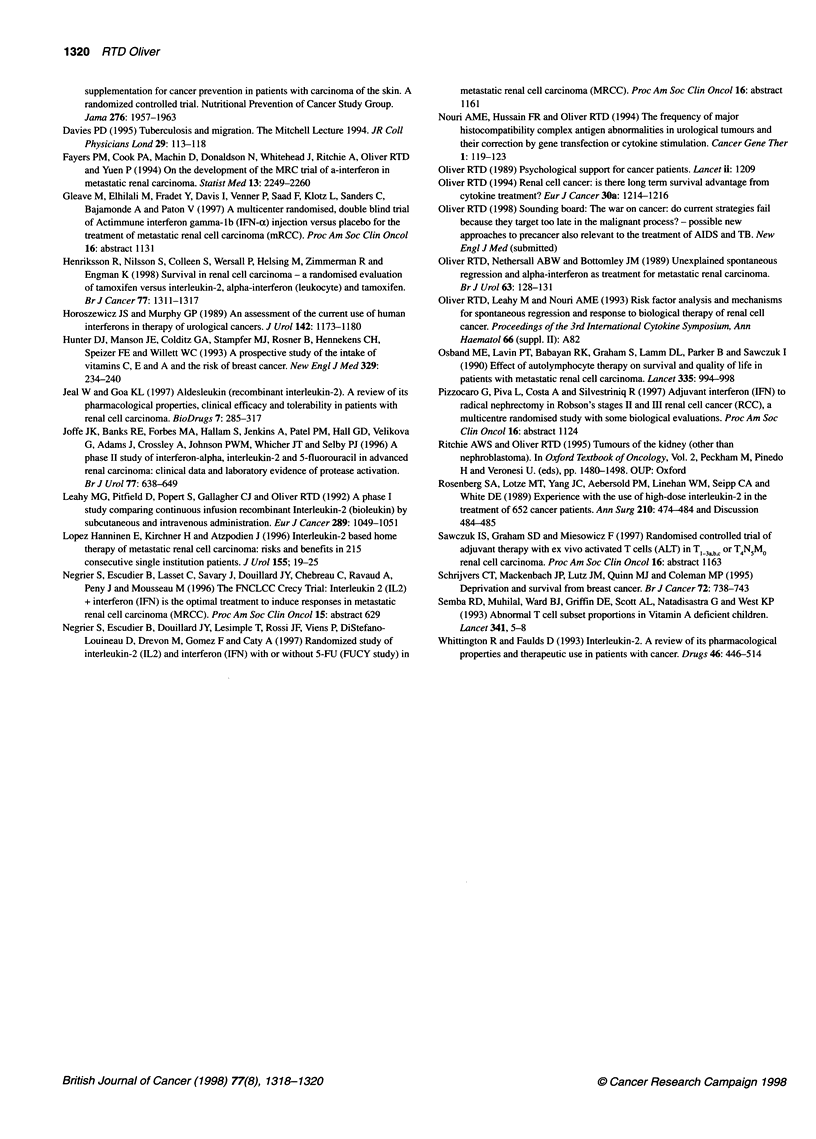# Are cytokine responses in renal cell cancer the product of placebo effect of treatment or true biotherapy? What trials are needed now?

**DOI:** 10.1038/bjc.1998.219

**Published:** 1998-04

**Authors:** R. T. Oliver

**Affiliations:** School of Medicine & Dentistry, St Bartholomew's Hospital, Department of Medical Oncology, West Smithfield, London, UK.


British Journal of Cancer (1998) 77(8), 1318–1320
© 1998 Cancer Research Campaign

Editorial

### Are cytokine responses in renal cell cancer the product of placebo effect of treatment or true biotherapy? What trials are needed now?

**RTD Oliver**

School of Medicine & Dentistry, St Bartholomew's and The Royal London Hospitals, Department of Medical Oncology, West Smithfield, London EC1A 7BE, UK

Today, my longest surviving patient with complete remission from single-agent interleukin 2 (IL-2) is alive and disease free 10 years after he had presented 2 years post nephrectopy with lung metastases and a renal bed recurrence (Leahy et al, 1992). This was invading the spinal canal and inducing severe pain that was poorly controlled even by high doses of morphine and had induced anaesthesia in the L2 dermatome. The fact that this patient was able to stop morphine within 7 days of starting treatment provides added credence to the reality of the response. Such observations make it difficult to believe that there is not a subgroup of patients with renal cell cancer that benefit from immune manipulation. This is despite the publication in this issue of yet another negative trial of biological therapy to go with the three others presented at ASCO this year. Once a true complete remission is achieved, relapse is rare and the chance of achieving such durable complete remission is linked to a specific HLA antigen in the responder (Bain et al, 1997), adding support to the idea that immune response is relevant. The debate today questions whether one achieves more response by combination therapy and why, repeatedly, the response rate to the same regimen has ranged so widely with initial encouraging response in the 30–40% range being followed by cummulative data indicating a 15–20% response (Oliver, 1994). The question arises as to whether there are other factors involved. Having attended rounds with Julian Bloom, whose response rate to medroxyprogesterone (17%) was 12% higher than that now reported (Bloom, 1973; Ritchie and Oliver, 1995) as well as with Steve Rosenberg whose initial response rate to IL-2 plus LAK cells (35%) was 12% higher than is now reported (Rosenberg et al, 1989; Whittington and Faulds, 1993), one has to accept either that there is a major selection in referral patterns to specialist centres or, having observed the powerful impact of these clinicians on patients, that there might be a placebo effect of treatment as a component of the response rate? Such concerns are the principal reason why, despite undisputed better survival in randomized trials after autologous lymphocyte therapy in both metastatic patients (Osband et al, 1990) and high-risk disease patients after nephrectomy (Sawczuk et al, 1997), there is scepticism in the scientific community as to whether it is a placebo effect of the process or whether the 6-weekly leucophoresis and retransfusing after in vitro lycophocyte activation by culture with cytokine 'soup' actually does alter immune response to renal cancer. The fact that survival correlates with IL-1 levels induced by the cytokine soup (Osband et al, 1990) is the main observation that allows one to believe that there might be a true immunological effect of therapy. In my own studies on spontaneous regression, the higher than expected incidence of events when initially published [7% after 64 cases studied (Oliver et al, 1989) vs 4% after 200 cases studied (Oliver et al, 1993)] may have reflected the fact that the first part of the series was treated before there was any serious therapy available for most of the time. Much of the consultation time was spent 'brainwashing' the patient that spontaneous regression was a realistic if rare event. The relief of psychiatric stress in four out of seven patients spontaneously regressing (Oliver, 1989) is witness to the fact that neuroendrocrine factors might make a contribution to response.

That selection is also a critical issue comes from the observation that there is an inverse relationship between the distance travelled for consultation and the frequency of response and survival in patients treated in our renal cell cancer studies. This included a patient who achieved an almost complete response after having had to travel to London from Sydney to get drug supplies as the drug is not available in Australia. This observation emphasizes how much patient selection could play a role. The virtual absence of response amongst renal cell cancer patients diagnosed in my own district, which has one of the highest levels of poverty in Europe, is the obverse of this observation. This is a particularly telling observation when taken together with the fact that breast cancer patients living in the same district have 20% worse survival than those patients diagnosed in outer London suburban districts (Oliver, 1997), whose renal cell cancer patients when treated by us have also had the highest frequency of response. That the same difference in survival is observed when patients from these extremes areas of residence are operated on for breast cancer by the same surgeons in my own hospital demonstrates that it is not an effect of delayed diagnosis or incompetent surgeons. These observations on the impact of poverty on cancer survival have been supported by the results of Shrijvers et al (1995) who also demonstrated that it was a factor even after correction for stage at presentation. Although the precise explanation for these differences is not known, they may reflect differences in environmental pollution. Alternatively, they may possibly reflect the effect of a lifetime exposure to subclinical deficiency of vitamin A, which is known to influence T-cell immune responses (Semba et al, 1993) and be a factor in breast cancer development (Hunter et al, 1993), and/or vitamin D deficiency, which is known to influence macrophage function (Davies, 1995) and be a factor in development of breast and prostate cancer (Clark et al, 1996).

It is against this background that one must view the randomized trial reported in this issue. This compares survival of 63 metastatic renal cell cancer patients receiving tamoxifen only vs 65 patients

**Table 1** Overview of cytokine phase 2 studies in metastatic renal cell cancer (Horoszewicz and Murphy, 1989; Jeal and Goa, 1997)

| | No. of cases | CR (%) | PR (%) | OR (%) |
|---|---|---|---|---|
| Interferon alpha alone | 1100 | 2 | 14 | 16 |
| IL-2 alone bolus | 404 | 6 | 10 | 16 |
| IL-2 alone c.i. | 444 | 4 | 11 | 15 |
| IL-2 alone s.c. | 146 | 3 | 21 | 24 |
| IL-2 + α-IFN i.v. | 121 | 7 | 18 | 25 |
| IL-2 + α-IFN s.c. | 513 | 6 | 15 | 21 |

CR, complete response; PR, partial response; OR, no response.

receiving tamoxifen plus interleukin 2/alpha interferon for 6 weeks followed by maintenance treatment for 5 days every 4 weeks for up to 12 months (Henriksson et al, 1998). This relatively small trial failed to show any significant survival benefit in this predominantly poor-risk population, despite a 3% complete response in the control arm and an 8% complete response in the treatment arm. This observation, taken with the three negative cytokine trials (Gleave et al, 1997; Negrier et al, 1997; Pizzocaro et al, 1997) reported at ASCO this year, raises the question as to whether there is any justification for further trials. More specifically, is there any justification for the proposed MRC/EORTC trial about to be launched comparing nothing vs IL-2/alpha interferon/5-FU, particularly as one of the negative trials reported at ASCO compared IL-2/alpha interferon ± 5-FU in metastatic disease (Negrier et al, 1997). The answer must be a resounding yes, and it could be said that there is a case for the drug companies involved to almost feel a moral obligation to encourage recruitment, given the continued ongoing scepticism about cytokine therapy. Two positive trials, the first involving 425 patients comparing interferon alone, interleukin 2 alone and the combination (Negrier et al, 1996) and the second comparing tamoxifen vs IL-2/alpha interferon/5-FU in patients with metastatic disease (Atzpodien et al, 1997) when progression-free survival was 13 months in 41 patients in the experimental arm and 4 months for 37 patients in the tamoxifen arm ($P$<0.01) are the strongest data to support the need for this trial. Easy access to ultrasound, leading to early diagnosis of renal tumours, and more frequent treatment of patients at a stage when they only have small-volume lung metastases with a high chance of response (Lopez Hanninen et al, 1996) are factors possibly explaining why the German data have for so long been felt to be so different from what can be achieved in other countries. This has been particularly so in the UK where most GPs have to wait 1–3 months for such a procedure to be performed and the response rate to the Atzpodian regimen has been half that seen in Germany (Joffe et al, 1996). The new MRC/EORTC trial is an adjuvant study that focuses on high-risk patients with vascular, lymphatic or local invasion (T3/T4) in complete remission after surgery. With evidence from the success of BCG in superficial bladder cancer but not advanced disease suggesting that immune response is more relevant at an early stage in the clonal evolution of cancer, the proposed trial gives the maximum opportunity for success.

However, even if this trial does succeed, as the paper of Henriksson et al (1998) indicates, there is a need for new approaches to working out dosages and duration of treatment with biological agents that have bell-shaped dose–response curves. Even for alpha interferon 20 years after it was first used, it is far from clear what is the optimum dose (Horoszewicz and Murphy, 1989). There has been some attempt to investigate this using in vitro studies on bladder cancer cell lines. These have suggested that beneficial alterations from cytokine treatment may only be detectable on 20% of tumours because 20% have absent HLA that cannot be induced and 60% had maximally expressed HLA that could not be up-regulated and only 20% showed up-regulation in response to interferon (Nouri et al, 1994). Before one could consider such an approach for use clinically, it would be necessary to develop a technique that could give an early result on fresh tumour samples. Had this been possible in Henriksson's trial, the exclusion of 80% of the cases and assumption that patients with complete responses have an 80% chance of durable survival would mean that the five complete responders in the IL-2/alpha interferon arm compared with the two in the tamoxifen arm would have translated into a 31% vs 12% 'cure,' which is nearly as good as the difference between bleomycin velbe and single-agent chemotherapy in testis cancer.

Although ideal in theory, developing and validating treatment response prediction assays would require substantive investment. Developing better approaches to producing more active combination therapy is more likely to be a better way forward. With the recently completed MRC trial of single-agent alpha interferon vs medroxyprogestereone acetate (Fayers et al, 1994) stopped and now undergoing detailed analysis after recruiting 365 patients in 5 years, there is an opportunity to examine the issue as to whether two or three drugs are better than one. A first step forward might be to confirm in a large randomized trial whether the observations in the original data of Atzpodien are correct (Lopez Hanninen et al, 1996), i.e. that in good and intermediate prognosis patients there is a 15% benefit from adding 5-FU, while at the same time doing a dose escalation study to confirm the literature overview data (Jeal and Goa, 1997) (see Table 1) that going from single-agent interleukin 2 or interferon to the two-drug combination of IL-2/alpha interferon is associated with a 10% improvement in all comers (Oliver, 1994). Setting up a large-scale pan-European trial with a 2 × 2 design aimed to recruit 1000 patients in 5 years to examine two doses of interferon ± interleukin 2 as one randomization and ± 5-FU as the second randomization in patients with metastatic renal cell cancer would provide really important information that would establish beyond doubt the place of biotherapy in this disease. Interest in this would be enhanced if it focused on incorporating continuous infusion 5-FU rather than bolus, as used by Atzpodien, as it has been shown to be better than bolus treatment in stomach cancer. Restricting entry to good- and intermediate-risk patients would leave the poor-risk patients for studies of new agents.

### REFERENCES

Atzpodien J, Kirchner H, Franzke A, Wandert T, Probst M, Buer J, Duensing S and Ganser A (1997) Results of a randomised clinical trial comparing SC interleukin-2, SC alpha-2a-interferon and IV bolus 5-fluorouracil against oral tamoxifen in progressive metastatic renal cell carcinoma patients. *Proc Am Soc Clin Oncol* **16**: abstract 1164

Bain C, Merrouche Y, Puisieux I, Blay JY, Negrier S, Bonadona V, Lasset C, Lanier F, Duc A, Gebuhrer L, Philp T and Favrot MC (1997) Correlation between clinical response to interleukin 2 and HLA phenotypes in patients with metastatic renal cell carcinoma. *Br J Cancer* **75**: 283–286

Bloom HJ (1973) Hormone-induced and spontaneous regression of metastatic renal cancer. *Cancer* **32**: 1066–1071

Clark LC, Combs GF Jr, Turnbull BW, Slate EH, Chalker DK, Chow J, Davis LS, Glover RA, Graham GF, Gross EG, Krongrad A, Lesher JL Jr, Park HK, Sanders BB Jr, Smith CL and Taylor JR (1996) Effects of selenium

© Cancer Research Campaign 1998

supplementation for cancer prevention in patients with carcinoma of the skin. A randomized controlled trial. Nutritional Prevention of Cancer Study Group. *Jama* **276**: 1957–1963

Davies PD (1995) Tuberculosis and migration. The Mitchell Lecture 1994. *JR Coll Physicians Lond* **29**: 113–118

Fayers PM, Cook PA, Machin D, Donaldson N, Whitehead J, Ritchie A, Oliver RTD and Yuen P (1994) On the development of the MRC trial of a-interferon in metastatic renal carcinoma. *Statist Med* **13**: 2249–2260

Gleave M, Elhilali M, Fradet Y, Davis I, Venner P, Saad F, Klotz L, Sanders C, Bajamonde A and Paton V (1997) A multicenter randomised, double blind trial of Actimmune interferon gamma-1b (IFN-α) injection versus placebo for the treatment of metastatic renal cell carcinoma (mRCC). *Proc Am Soc Clin Oncol* **16**: abstract 1131

Henriksson R, Nilsson S, Colleen S, Wersall P, Helsing M, Zimmerman R and Engman K (1998) Survival in renal cell carcinoma – a randomised evaluation of tamoxifen versus interleukin-2, alpha-interferon (leukocyte) and tamoxifen. *Br J Cancer* **77**: 1311–1317

Horoszewicz JS and Murphy GP (1989) An assessment of the current use of human interferons in therapy of urological cancers. *J Urol* **142**: 1173–1180

Hunter DJ, Manson JE, Colditz GA, Stampfer MJ, Rosner B, Hennekens CH, Speizer FE and Willett WC (1993) A prospective study of the intake of vitamins C, E and A and the risk of breast cancer. *New Engl J Med* **329**: 234–240

Jeal W and Goa KL (1997) Aldesleukin (recombinant interleukin-2). A review of its pharmacological properties, clinical efficacy and tolerability in patients with renal cell carcinoma. *BioDrugs* **7**: 285–317

Joffe JK, Banks RE, Forbes MA, Hallam S, Jenkins A, Patel PM, Hall GD, Velikova G, Adams J, Crossley A, Johnson PWM, Whicher JT and Selby PJ (1996) A phase II study of interferon-alpha, interleukin-2 and 5-fluorouracil in advanced renal carcinoma: clinical data and laboratory evidence of protease activation. *Br J Urol* **77**: 638–649

Leahy MG, Pitfield D, Popert S, Gallagher CJ and Oliver RTD (1992) A phase I study comparing continuous infusion recombinant Interleukin-2 (bioleukin) by subcutaneous and intravenous administration. *Eur J Cancer* **289**: 1049–1051

Lopez Hanninen E, Kirchner H and Atzpodien J (1996) Interleukin-2 based home therapy of metastatic renal cell carcinoma: risks and benefits in 215 consecutive single institution patients. *J Urol* **155**; 19–25

Negrier S, Escudier B, Lasset C, Savary J, Douillard JY, Chebreau C, Ravaud A, Peny J and Mousseau M (1996) The FNCLCC Crecy Trial: Interleukin 2 (IL2) + interferon (IFN) is the optimal treatment to induce responses in metastatic renal cell carcinoma (MRCC). *Proc Am Soc Clin Oncol* **15**: abstract 629

Negrier S, Escudier B, Douillard JY, Lesimple T, Rossi JF, Viens P, DiStefano-Louineau D, Drevon M, Gomez F and Caty A (1997) Randomized study of interleukin-2 (IL2) and interferon (IFN) with or without 5-FU (FUCY study) in metastatic renal cell carcinoma (MRCC). *Proc Am Soc Clin Oncol* **16**: abstract 1161

Nouri AME, Hussain FR and Oliver RTD (1994) The frequency of major histocompatibility complex antigen abnormalities in urological tumours and their correction by gene transfection or cytokine stimulation. *Cancer Gene Ther* **1**: 119–123

Oliver RTD (1989) Psychological support for cancer patients. *Lancet* **ii**: 1209

Oliver RTD (1994) Renal cell cancer: is there long term survival advantage from cytokine treatment? *Eur J Cancer* **30a**: 1214–1216

Oliver RTD (1998) Sounding board: The war on cancer: do current strategies fail because they target too late in the malignant process? – possible new approaches to precancer also relevant to the treatment of AIDS and TB. *New Engl J Med* (submitted)

Oliver RTD, Nethersall ABW and Bottomley JM (1989) Unexplained spontaneous regression and alpha-interferon as treatment for metastatic renal carcinoma. *Br J Urol* **63**: 128–131

Oliver RTD, Leahy M and Nouri AME (1993) Risk factor analysis and mechanisms for spontaneous regression and response to biological therapy of renal cell cancer. *Proceedings of the 3rd International Cytokine Symposium, Ann Haematol* **66** (suppl. II): A82

Osband ME, Lavin PT, Babayan RK, Graham S, Lamm DL, Parker B and Sawczuk I (1990) Effect of autolymphocyte therapy on survival and quality of life in patients with metastatic renal cell carcinoma. *Lancet* **335**: 994–998

Pizzocaro G, Piva L, Costa A and Silvestriniq R (1997) Adjuvant interferon (IFN) to radical nephrectomy in Robson's stages II and III renal cell cancer (RCC), a multicentre randomised study with some biological evaluations. *Proc Am Soc Clin Oncol* **16**: abstract 1124

Ritchie AWS and Oliver RTD (1995) Tumours of the kidney (other than nephroblastoma). In *Oxford Textbook of Oncology*, Vol. 2, Peckham M, Pinedo H and Veronesi U. (eds), pp. 1480–1498. OUP: Oxford

Rosenberg SA, Lotze MT, Yang JC, Aebersold PM, Linehan WM, Seipp CA and White DE (1989) Experience with the use of high-dose interleukin-2 in the treatment of 652 cancer patients. *Ann Surg* **210**: 474–484 and Discussion 484–485

Sawczuk IS, Graham SD and Miesowicz F (1997) Randomised controlled trial of adjuvant therapy with ex vivo activated T cells (ALT) in $T_{1-3a,b,c}$ or $T_4N_5M_0$ renal cell carcinoma. *Proc Am Soc Clin Oncol* **16**: abstract 1163

Schrijvers CT, Mackenbach JP, Lutz JM, Quinn MJ and Coleman MP (1995) Deprivation and survival from breast cancer. *Br J Cancer* **72**: 738–743

Semba RD, Muhilal, Ward BJ, Griffin DE, Scott AL, Natadisastra G and West KP (1993) Abnormal T cell subset proportions in Vitamin A deficient children. *Lancet* **341**, 5–8

Whittington R and Faulds D (1993) Interleukin-2. A review of its pharmacological properties and therapeutic use in patients with cancer. *Drugs* **46**: 446–514

© Cancer Research Campaign 1998